# Seasonal association between ambient ozone and hospital admission for respiratory diseases in Hanoi, Vietnam

**DOI:** 10.1371/journal.pone.0203751

**Published:** 2018-09-24

**Authors:** Ly M. T. Luong, Dung Phung, Tran Ngoc Dang, Peter D. Sly, Lidia Morawska, Phong K. Thai

**Affiliations:** 1 Faculty of Medicine, The University of Queensland, Brisbane, Australia; 2 Children's Health and Environment Program, The University of Queensland, Brisbane, Australia; 3 Faculty of Environmental Sciences, VNU University of Science, Hanoi, Vietnam; 4 Centre for Environment and Population Health, Griffith University, Brisbane, Australia; 5 The Institute of Research and Development, Duy Tan University, Da Nang, Vietnam; 6 Department of Environmental Health, University of Medicine and Pharmacy, Ho Chi Minh City, Vietnam; 7 International Laboratory for Air Quality & Health, Queensland University of Technology, Brisbane, Australia; Ospedale S. Corona, ITALY

## Abstract

**Background:**

Many studies have indicated the detrimental effect of ambient ozone to respiratory health in different countries. The levels of ozone in Hanoi, Vietnam are frequently above the WHO guideline but very few studies on the effects of ambient ozone on human health have been conducted in this location. This study aimed to examine the effects of ozone on hospital admission for respiratory diseases in Hanoi, by diseases, ages and seasons.

**Methods:**

Hospital admissions, air pollutants and meteorological data were collected from January 2010 to June 2014. We used generalized linear models and distributed lag linear model to assess the association. In addition to full year analysis, we conducted restricted analysis of the data for two summer (from June-August) and winter (from December-February) seasons and grouped hospital admissions by diseases and ages (all ages, children 0 to 5 years and elderly >65 years). The delayed effect of ozone was assessed using lags of up to 5 days.

**Results:**

Ozone has a stronger effect on the risk of hospital admission for respiratory diseases and wheeze-associated disorders in the winter. For respiratory diseases, children were affected by ozone more than other age groups in both winter and summer. Each increase of 10 μg/m^3^ of ozone is associated with an increase of 6.2% risk of admission for respiratory disease among children in the winter and 1.2% in the summer. For wheeze-associated disorders, the elderly group seemed to be more affected by ozone in full year and winter but no significant association was found between ozone and admission for wheeze-associated diseases in any age group.

**Conclusions:**

Ozone is a risk factor for respiratory admission, especially amongst children under 5 years old in Hanoi, and ozone has a stronger effect in the winter than in the summer in this city.

## Introduction

Long-term monitoring of air quality indicates that air pollution levels are rising in many cities throughout the world [1]. Among the criteria air pollutants, ground-level ozone (O_3_) commonly exceeds the recommended upper limit in many parts of the world, and is generally increasing in East Asia [2–4].

The strong oxidant, O_3_, has been reported to associate with adverse effects on human health including respiratory, cardiovascular diseases or even premature death [5, 6]. Acute exposure to air with high levels of O_3_ can trigger chest pain, cough and wheeze, throat irritation, and airway inflammation. Longer term exposure can reduce lung function and lung growth, lead to the development of asthma, and induce exacerbations of bronchitis, chronic obstructive pulmonary disease (COPD) and asthma [6]. Increased ground-level O_3_ has been associated with asthma and respiratory-related exacerbations [5, 7–11]. However, not all studies report these associations [12–14]. Clarifying associations between O_3_ and respiratory ill health, especially in ethnically and geographically diverse populations, is important as wheeze-associated disorders and asthma are common causes of hospitalization in children [11].

Young children, especially children younger than 5 years old, are more sensitive to air pollutants than adults; for a variety of reasons, including; their smaller airways; higher breathing rate and minute ventilation relative to body size; immature and developing lungs and immune systems; and they spend more time outdoors exposed to ambient air. [9, 15]. The elderly (> 65 years old) are also most susceptible to air pollution, with an increased need for clinic visits for respiratory illnesses [16–18].

Most of the studies in the literature come from North America, Europe or China [19] although the burden of air pollution is heavy in the developing countries of South and South East Asia [20]. Vietnam is one of the countries where rapid economic expansion and urbanization has been associated with serious air pollution [21]. The levels of air pollutants in Hanoi are frequently above the WHO guidelines for both particulate and gaseous pollutants including O_3_ [21]. According to the data from air quality monitoring stations, the highest level of ozone recorded in Hanoi was 455 μg/m^3^ (1-hour mean) and 346 μg/m^3^ (8-hour mean) which was 3.5 times higher than the WHO suggested level for O_3_ (100 μg/m^3^ 8-hour mean) [22]. Major contributors to the high level of ozone and other air pollutants in Hanoi are the increasing number of road vehicles, crop stubble burning in suburban areas, and the effects of fossil fuel power plants in the neighbouring provinces.

Daily mean O_3_ values vary across seasons, increasing from spring to reach peak values during summer, with a gradual decline into winter troughs [6, 23]. Some studies have reported a stronger association between ambient O_3_ and adverse health outcomes in summer while others found a more pronounced association during the cold season [19, 24–28].

While some studies have examined the effects of ambient air pollution on various health outcomes in Vietnam, [29–31], only one has evaluated the effect of O_3_ on respiratory disease [32]. The present study aims to examine the association between ambient O_3_ and respiratory-related hospitalisations, especially among children and the elderly, in Hanoi in two different seasons, winter and summer.

## Materials and methods

### Research location

The study was conducted in Hanoi, which has a population of about 7 million with a density of more than 2000 people/ km^2^. Children aged < 5 years old and the elderly aged >65 years old accounted for 9% and 8%, respectively, of the total population of the city. The annual population growth rate was 2% [33].

During 2013, Hanoi’s economy grew by 8%, significantly higher than the national average of 5.3% [34, 35]. Hanoi has seen a large increase in the number of road vehicles, second only to Ho Chi Minh City in Vietnam. During the ten years from 2003 to 2013, the number of cars and motorbikes in Hanoi increased from 1 million to 5 million vehicles [34, 36].

### Data collection

#### Hospital admissions

Data on daily hospital admissions from January 2010 to June 2014 were collected from three hospitals in Hanoi, the Vietnam National Hospital of Paediatrics (Paediatrics) and two multi-department hospitals, Bach Mai (BM) and Duc Giang (DG) hospitals. Data extracted included age, address, date of admission and the International Classification Diseases 10th revision (ICD10) code. Patients who came from locations other than Hanoi were excluded. We grouped hospitalizations as: all-cause respiratory diseases (ICD10 code: J00-J99) and wheeze-associated disorders (ICD10 code: J21, J22, J45, J46). We examined the effects of O_3_ on all ages group including patients at any age and 2 specific age groups including children and the elderly. Children included all patients aged from 28 days to less than 5 years old. Neonatal admissions (aged 0–27 days) were excluded as they are likely to be affected by perinatal conditions [37]. The elderly included all patients aged > 65 years old.

#### Ozone and meteorological data

Ozone and meteorological data (hourly values) were obtained from the Centre for Environmental Monitoring Portal (Vietnam Environment Administration) from January 2010 to June 2014. The data were recorded from a national automatic air quality monitoring station in Hanoi. Daily average temperature (°C), relative humidity (%) and daily average concentration of O_3_ (μg/m^3^) were calculated from collected hourly values. A 75% completeness criterion was applied to calculate daily aggregate data, meaning that if less than 18 hours of temperature, relative humidity and O_3_ concentration data were available in a day then the daily average concentration for the day was assigned as ‘missing’ data. All missing values were replaced by the value generated by the mean- before-after method for 1-day gap and the multiple imputation method with linear regression for continuous variables for longer gaps [38, 39].

### Data analysis

We performed a time series regression analysis to examine the relationship between daily concentrations of O_3_ and hospital admissions due to respiratory diseases and wheeze-associated disorders. We used generalized linear models and distributed lag linear model with the family of quasi-Poisson distribution to assess the effect of daily O_3_ on respiratory admissions while adjusting for the effect of temperature and humidity [40–42].

Our model included two cross-basis matrices. The first cross-basis for O_3_ comprised a linear function for the space of the predictor. The delayed effect of O_3_ was assessed using lags of up to 5 days. The second cross-basis was for daily temperatures, applying a quadratic B-spline function with 3 strata intervals at lags of 0–1, 2–5, 6–10 days. The maximum lag for temperature was set to 10 days [41–43].

A smooth function of time with 7 degree of freedom (df) per year was included in the regression models to correct for seasonality and long-time trend [41]. Time variable is a series of equally-spaced point taken each day in time order during the period of study. In order to adjust for the effects of relative humidity, we used a natural spline function of daily average humidity with 3 df. In addition, we also added the day of the week (DOW) into the model to adjust for the potential DOW’s effect on hospital admissions (Eq 1)

To estimate the effect of ozone on hospital admission in hot and cold weather separately, we also restricted analysis of the data to two specific seasons: summer (from June-August) and winter (from December–February) in addition to the full year analysis. We used natural splines for day of the year with 4 df and time with 3 df in the regression models to describe the seasonal effect within each year and the long-time trend, respectively (Eq 2) [41].

The final models are shown below:

$$Y_{t}∼quasi-Poisson\;(\mu_{t})$$ (Eq 1)

$$Ln(\mu_{t})=\alpha +{cb}_{Oz}({Oz}_{t},\ldots ,{Oz}_{t-5})+{cb}_{T}(T_{t},\ldots ,T_{t-10})+ns(time,5*7\;df)+ns\;(RH,3df)+\gamma DOW$$

$$Y_{t}∼quasi-Poisson(\mu_{t})$$ (Eq 2)

$$Ln(\mu_{t})=\alpha +{cb}_{Oz}({Oz}_{t},\ldots ,{Oz}_{t-5})+{cb}_{T}(T_{t},\ldots ,T_{t-10})+ns(doy,4df)+ns(time,3\;df)+ns(RH,3df)+\gamma DOW$$

Where *Y_t_* is the observed daily count of hospital admissions (respiratory and wheeze-associated disorders) on day t; α is the intercept; *cb_Oz_* is the cross-basic for O_3_; *Oz_t_* is daily average concentration of O_3_ on day t; *cb_T_* is the cross-basic for temperature; *T_t_* is the daily average temperature on day t; ns is a natural cubic spline function; *doy* is day of the year; *time* is variable running from 1 to 1642; *RH* is the daily average humidity; *DOW* is the categorical day of the week with a reference day of Sunday.

Autocorrelation in the residuals was checked for and pre-removed before analysing in the time series models. A sensitivity analysis using DLNM with a threshold (mean concentration of O3 in winter and summer) was run to check if there is any significantly different outcomes from our non-threshold approach. Results of sensitivity analysis are presented as supplementary material (S1–S3 Tables).

All statistical analyses were performed using R software version 2.3.2 (http://www.r-project.org), using the “dlnm”, “mvmeta”, “splines”, “tsModel” and “lubridate” packages. The results are presented as the Risk Ratio (RR) and its 95% confidence interval (CI) for daily respiratory hospital admissions, per 10 μg/m^3^ increase in O_3_ at single lag days and cumulative lag of 5 days, by using STATA 12 (Stat Corporation, College Station, Texas, USA).

The ethical clearance of this study was approved by the Vietnam National Hospital of Paediatrics, Biomedical Research Ethics Committee (NHP–RICH– 15–014) and UQ School of Medicine Low Risk Ethical Review Committee (#2016-SOMILRE-0155). This ethical clearance allows us to use the hospital admission data (includes age, province level address, date of admission and the ICD10 code), which have been de-identified, in our research. As the data are anonymous, we are not required to obtain informed written consent from patients (or their parents/guardians in the case of minors).

## Results

A total of 92,183 hospital admissions for respiratory diseases were recorded during the study period in the three participating hospitals in Hanoi (Table 1), with 78% for children aged <5 years old and 10% for the elderly aged >65 years old. Wheeze-associated disorders accounted for 10,031 admissions of all ages combined (about 11% of the all cause respiratory diseases). Admissions among children and the elderly made up 69% and 7%, respectively.

**Table 1:** Descriptive statistics of hospitalisation, air pollution and meteorological variables.

	Percentile			Minimum	Maximum	Mean (SD)
	25th	50th	75th			
**Daily hospital admissions**						
**All ages**						
All causes of respiratory diseases (92,183 admissions)						
	43	55	67	13	121	56 (18)
Wheeze-associated disorders (10,031 admissions)						
	4	6	8	0	23	6 (3)
**Children (<5 years old)**						
All causes of respiratory diseases (66,685 admissions)						
	31	39	49	10	92	41 (14)
Wheeze-associated disorders (6,902 admissions)						
	2	4	6	0	19	4(3)
**The elderly (>65 years old)**						
All causes of respiratory diseases (9,544 admissions)						
	3	5	8	0	19	6 (3)
Wheeze-associated disorders (698 admissions)						
	0	0	1	0	3	0.4 (0.7)
**Air pollutants (**μ**g/m**^**3**^**) and meteorological variables**						
**Full year average**						
24-hour mean O_3_ (μg/m^3^)	28.9	41.8	57.8	0.1	196.6	46.9 (28.6)
1-hour mean O_3_ (μg/m^3^)	15.4	31	63.8	0.0	455	50.2 (53.9)
Temperature (°C)	20.1	25.7	29.1	9.6	35.9	24.5 (5.8)
Relative humidity (%)	71.5	78.3	85.3	39.8	99.4	78.2 (10.7)
**Winter average**						
24-hour mean O_3_ (μg/m^3^)	30.9	40.3	51.4	4.1	115.4	42.4 (17.5)
Temperature (°C)	14.2	17	20	9.6	26.8	17.4 (3.7)
**Summer average**						
24-hour mean O_3_ (μg/m^3^)	25.5	41	69.4	7.3	196.6	52 (37.6)
Temperature (°C)	28.7	30.1	31.5	18.5	35.9	30.1 (2.2)

SD: Standard Deviation

The average daily mean level of O_3_ was 46.9 μg/m^3^ over the entire study period, 42.4 μg/m^3^ in winter, and 52 μg/m^3^ in summer (Table 1). There were 193 days (46%) in the winter where the daily mean of O_3_ was above 42.4 μg/m^3^ and 190 days (45.2%) in the summer where it was above 52 μg/m^3^. The maximum level for 24-hour mean O_3_ was 196.6 μg/m^3^ and for 1-hour mean O_3_ was recorded at 455 μg/m^3^ which was more than twice of the Vietnam national air quality standard level (200 μg/m^3^). During the study period, the O_3_ levels in Hanoi exceeded the Vietnam air quality guideline for O_3_ 1-hour mean (200 μg/m^3^) in nearly one thousand occasions (on more than 70 days) and exceeded the 1-hour mean value suggested by WHO (100 μg/m^3^) in more than 3700 occasions (on more than 350 days).

The average daily mean temperature in Hanoi during the study period was 24.5° C with a minimum of 9.6° C and a maximum of 35.9° C. In the winter, the average temperature was 17.4° C, and 30.1° C in summer. The average daily mean relative humidity was 78.2%, with a minimum of 39.8% and maximum of 99.4% (Table 1).

**Fig 1:**
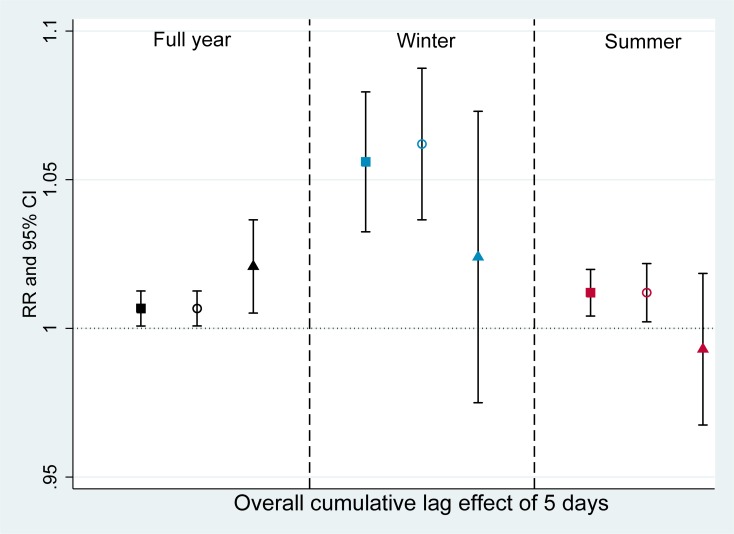
Association between O_3_ and hospital admissions for respiratory diseases (Overall cumulative lag effect of 5 days). RR: risk ratio, CI: confidence interval. Black square: full year, all ages; Blue square: winter, all ages; Red square: summer, all ages. Black hollow circle: full year, <5 years old; Blue hollow circle: winter, <5 years old; Red, hollow circle: summer, <5 years old. Black triangle: full year, >65 years old, Blue triangle: winter, >65 years old; Red triangle: summer, >65 years old.

Fig 1 presents the overall effects of O_3_ on hospital admission for respiratory diseases. The overall cumulative lag effect of 5 days exposure to O_3_ on hospital admission was statistically significant in each age groups. An increase of 10 μg/m^3^ in O_3_ level was associated with an increase of 0.7% (95% CI 0.1%– 1.3%) risk of admissions for respiratory diseases among both all ages and children group and of 2.1% (95% CI 0.5%-3.7%) among elderly group. When the data were restricted to specific seasons, in the winter, RR for respiratory admission was highest among children aged < 5 year old as compared to all ages and the elderly. For instance, the RR for each 10 μg/m^3^ increase of O_3_ was highest among children group (RR = 1.062, 95%CI 1.037–1.088) in the winter and lower in all ages (RR = 1.056, 95%CI 1.034–1.079) and the elderly group (RR 1.024, 95%CI 0.977–1.073). A similar pattern was also found in the summer for each age group but the effect of O_3_ was clearly weaker than in the winter, especially among children and all ages group which were (RR 1.012, 95%CI 1.003–1.021) and (RR 1.012, 95% CI 1.004–1.020), respectively. For elderly group, we did not observe statistically significant effect of O_3_ in specific season (Fig 1, S1 Table).

**Fig 2:**
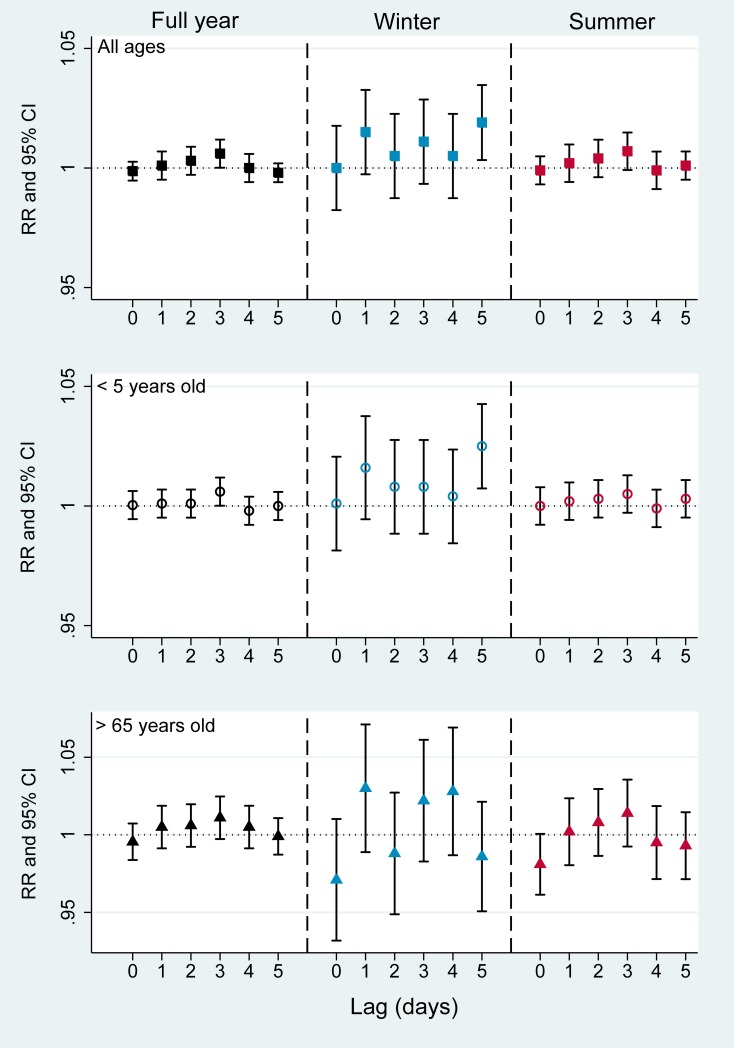
Associations between O_3_ and hospital admissions for respiratory diseases by seasons and age groups (Single day lag effect). RR: risk ratio, CI: confidence interval. Black square: full year, all ages; Blue square: winter, all ages; Red square: summer, all ages. Black hollow circle: full year, <5 years old; Blue hollow circle: winter, <5 years old; Red, hollow circle: summer, <5 years old. Black triangle: full year, >65 years old, Blue triangle: winter, >65 years old; Red triangle: summer, >65 years old.

Fig 2 presents single day lag effects of O_3_ on hospital admission for respiratory diseases by seasons and age groups. In the full year model, O_3_ tended to increase its effect after exposure at lag 0 to the strongest effect at lag 3 and decreased after that. At lag 3, the RR for each 10 μg/m^3^ increased of O_3_ was 1.006 (95%CI 1.001–1.011), 1.006 (95%CI 1.001–1.012) and 1.011 (95%CI 0.998–1.024) for all ages, children and the elderly group, respectively. A similar pattern with equivalent RRs was found with the summer models for each age groups but the O_3_ effects in the summer failed to reach statistical significance. In the winter, the effects of O_3_ significantly stronger than those in the summer or full year. Statistically significant associations were observed at a lag of 5 days among all ages (RR 1.019, 95% CI 1.003–1.036) and children aged <5 years old (RR 1.025, 95% CI 1.007–1.044). Ozone had a greater effect on children group compared to others (Fig 2, S1 Table).

**Fig 3:**
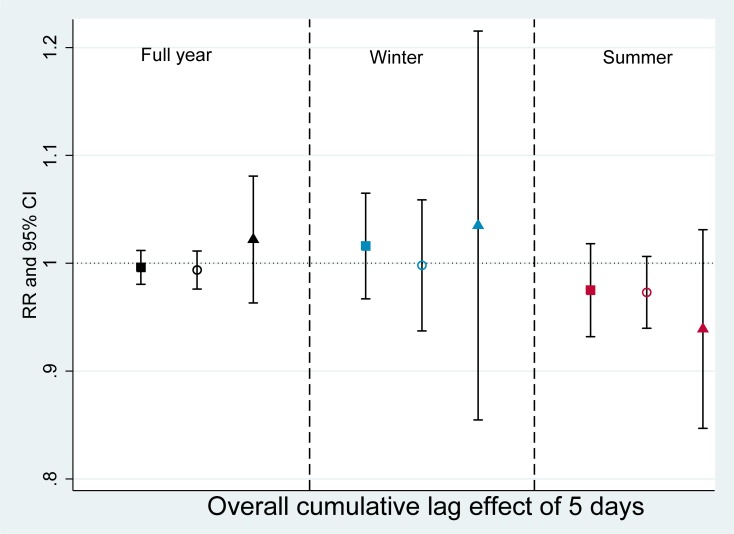
Association between O_3_ and hospital admissions for wheeze-associated disorder (Overall cumulative lag effect of 5 days). RR: risk ratio, CI: confidence interval. Black square: full year, all ages; Blue square: winter, all ages; Red square: summer, all ages. Black hollow circle: full year, <5 years old; Blue hollow circle: winter, <5 years old; Red, hollow circle: summer, <5 years old. Black triangle: full year, >65 years old, Blue triangle: winter, >65 years old; Red triangle: summer, >65 years old.

**Fig 4:**
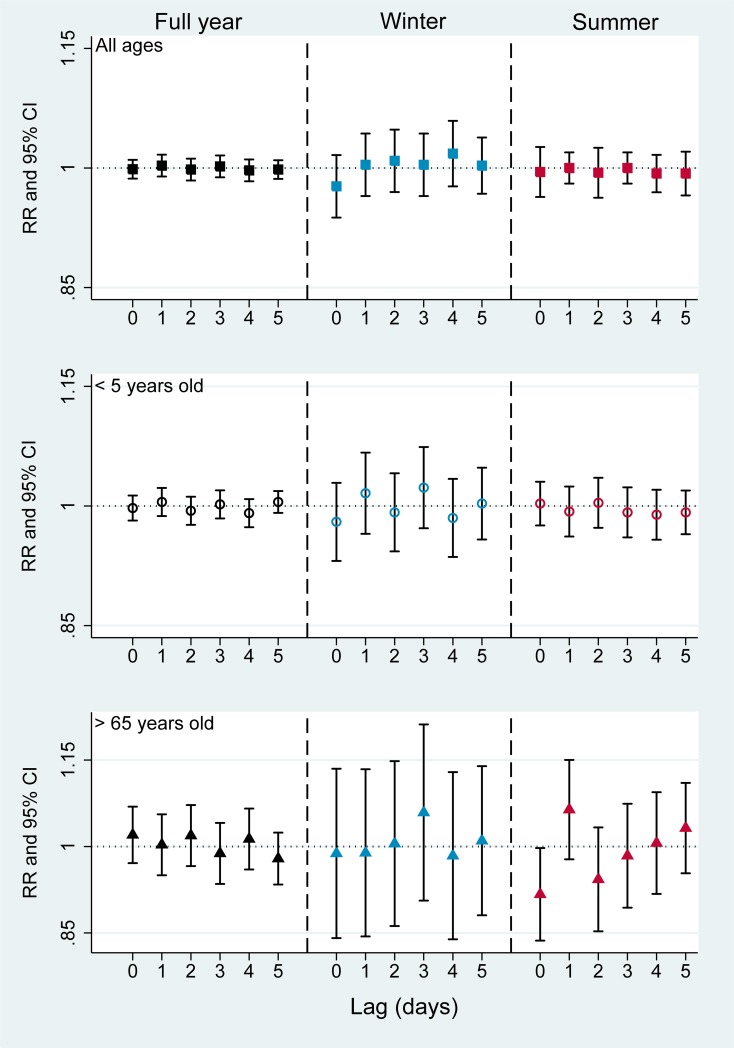
Associations between O_3_ and hospital admissions for wheeze-associated disorder by seasons and age groups (Single day lag effect). RR: risk ratio, CI: confidence interval. Black square: full year, all ages; Blue square: winter, all ages; Red square: summer, all ages. Black hollow circle: full year, <5 years old; Blue hollow circle: winter, <5 years old; Red, hollow circle: summer, <5 years old. Black triangle: full year, >65 years old, Blue triangle: winter, >65 years old; Red triangle: summer, >65 years old.

Figs 3 and 4 show that O_3_ had some positive but non-statistically significant effects on admission for wheeze-associated disorders. The RR for overall cumulative association after 5 days exposure were estimated highest among elderly group in the full year model (RR = 1.021, 95%CI 0.966–1.082) and the winter model (RR = 1.035, 95% CI 0.880–1.216) but lowest in the summer model (RR = 0.939, 95%CI 0.856–1.031) (Fig 3, S2 Table).

Regards to delayed effects of O_3_, the highest effects were observed in the winter and higher in elderly group. For instance, the greatest effect was estimated at lags 4 in all ages (RR = 1.018, 95% CI 0.979–1.059), lag 3 in children (RR = 1.023, 95% CI 0.975–1.074) and lag 3 among elderly (RR = 1.059, 95% CI 0.925–1.213). Ozone in summer seems to have lowest effect on wheeze-associated disorders but we observed a very high positive effect of O_3_ on elderly group at lag 1 (RR = 1.064, 95% CI 0.983–1.150). However, no statistically significant effects were found for O_3_ on the admissions for wheeze-associated disorders in any age group or season (Fig 4, S2 Table).

## Discussion

This present study is amongst the first to examine the short-term effect of air pollution on the risk of hospital admission in Hanoi, Vietnam with specific focus on the relationship between ground-level ozone and daily hospital admission for respiratory diseases and wheeze-associated disorders.

Although there is abundance of peer-reviewed published literature supporting an association between ambient ozone concentrations and adverse health effects so far, the most recent Integrated Science Assessment (ISA) document of the US EPA on ozone also showed that the evidence of ozone effect is mixed [6]. The ISA also overlooked the Zemp et al. (1999) study [13] from a large cohort study in Europe whose main conclusion was no association between ozone and respiratory symptoms. Additionally, a review paper on air pollution and health [44] has pointed out that there were “large inter individual differences in responsiveness to inhaled ozone”, which was reflected in the ISA as well. Therefore, our study with the aim of evaluating and quantifying the impact of ambient ozone level on the local population with different socio-economic conditions than the US is well justified.

The data from the present study show that there was a positive effect of ozone on the risk of hospital admissions for respiratory diseases, especially amongst young children. Ozone showed significant cumulative effects on hospitalization of children and all ages in both winter and summer. These results are in agreement with some previous studies in other parts of the world which reported significant increases in respiratory admission due to increases in the level of O_3_ [45, 46]. In the present study, O_3_ increased the risk of respiratory-related hospitalisations, with the maximum effect at 5-day lag between exposure and effect, especially in winter. While this was significant for all ages and for children <5 years old, the all age-effects are likely to be primarily due to effects on children, as evidenced by the high risk ratio for children and the lack of effect on the older adults.

Previous studies also observed significant effects of ozone on total respiratory disease among patients of all ages [47–50] and among children or the elderly [10, 51, 52] at some specific lags. For example, in one study conducted in Canada, the authors found respiratory admissions were associated with elevated ozone levels at 2, 3, 4, and 5 days prior to admission with the strongest association observed at a lag of 4 days, and with the odds ratio for children aged <3 years old of 1.22 (95% CI: 1.15–1.30) and for the elderly of 1.13 (1.09–1.18), based on an increment in ozone level of 19 μg/m^3^ [10]. Similarly, Vahedian et al. (2017) reported in their study that O_3_ showed a negative association with respiratory hospital admissions among all ages at lag 0 but a positive association at lag 1 day with a corresponding RRs (95% CI) of 1.010 (1.002–1.020) per 10 μg/m^3^ increase [50]. However, in another study, Fusco et al (2001) found O_3_ was associated with admissions only among children (0-14yo) (lag 1, 5.5% increase per IQR, 23.9 μg/m^3^) for total respiratory admissions while no association was observed for all ages.

Not all studies in the literature find positive associations between O_3_ and hospital admissions for wheeze-associated disorders [32, 51, 53, 54]. In a study conducted in Italy, Fusco et al. (2001) found no significant effect of ozone on admission for asthma among all ages (RR: 1.038, 95% CI: 0.997–1.011) and a negative effect for children aged <14 years old (RR: 0.984, 95% CI: 0.887–0.909). Another study in Malaysia did not observe significant associations between ozone and admissions for acute bronchitis (RR = 1.002, 95% CI 1.000–1.004), acute bronchiolitis (RR = 0.990, 95% CI 0.978–1.002) or asthma (RR = 1.000, 95% CI 0.999–1.000) among children < 5 years old [54]. In our previous study in Ho Chi Minh city, Vietnam, we did not observe any significant association between O_3_ and respiratory hospitalizations for any age groups (<5 years old, all ages and >65 years old) [31], not did a recent Vietnamese study with hospital admission for bronchitis and asthma (RR = 1.003, 95% CI 0.903–1.114) among children aged 1–5 years old [32].

Few studies have specifically accounted for seasonal differences in O_3_. Positive associations with respiratory admissions have been reported in summer only [45, 47], the warm season [48], or in the winter only [55]. In the present study, we observed positive associations with a 5-day cumulative lag in both winter and summer months but single day lag effects were only seen in winter. The reasons for these inconsistent findings is not clear but may be contributed to by: the absolute level of ambient ozone, differing patient characteristics (such as age, sex, occupation or poverty), the amount of outdoor activity undertaken and/or any adaptive behaviours (such as the use of open windows or using air conditioning), which can differ by location [17, 56]. The average mean and maximum concentration of ozone in Hanoi is generally higher than in Ho Chi Minh city [31]. In Vietnam, people often avoid going out when it is hot and sunny, when the photochemical formation of O_3_ from NO_2_ is maximal. Therefore, despite the higher ozone concentration during summer in Hanoi the adverse effects on health were less than those seen in winter. Another possible reason for inconsistent reports of seasonal effects may be insufficient adjustment for temperature [57]. Previous studies did not adequately control for temperature using the same day lags as for O_3_ [19]. In addition, since ozone has been receiving increasing attention over the past 30 years, associations between ozone and hospitalizations may have changed over this time frame due to changes in socioeconomic factors which vary by region [56]. An increase in the use of air conditioning, which is related to socioeconomic status, can modify exposure to ozone [56, 58].

### Limitations

We acknowledge that there were some limitations to this study. First, admission data were obtained from 3 hospitals which are not at the same level. Paediatrics and BM are national hospitals with 1300 and 1400 beds, respectively, while DG is a regional hospital, with 510 beds. However, Paediatrics is the tertiary level hospital for children while BM and DG are the general hospital which receive patients of all ages. Due to their heterogeneous characteristics, we could not check their effects and assume there is no hospital effects in our study. Second, the ambient monitoring data we used were collected from the only functioning monitoring station in Hanoi, which may not be representative of the whole city and it is possible that the effects of ozone might be under or overestimated. In addition, using imputation methods to replace missing ozone data may have introduced bias to the results of our analyses although the imputation amount is minimal. Third, data of other pollutants (particulate matters, nitrogen dioxide, sulphur dioxide, carbon monoxide) and other environmental variables (rainfall, wind speed) were not controlled in the models to see if they may attenuate the ozone effect as they were not available for the entire study period. Next, no data on individual exposure to ambient ozone or information on adaptive measures (e.g. air- conditioning usage, outdoor activity) were available in Vietnam which might be factors that we could not adjust in our models. Finally, the short time frame of this study might hinder the possibility of analysing the effects of all confounding factors to the impact of ozone on respiratory admissions.

## Conclusions

The findings of this study demonstrated that ozone was associated with an increased risk of respiratory-related admissions, especially for children under the age of 5 years. The effect was stronger in the winter than in the summer in each age group. In the winter, for each increase of 10 μg/m^3^ of O_3_, the risk of admissions for respiratory diseases after 5 days of exposure increased 6.2% (95% CI 3.7%– 8.8%) among children, 5.6% (95% CI 3.4%-7.9%) for all ages. No significant association between O_3_ and hospital admission for wheeze-associated disorders was found for any age. These findings suggested that O_3_ is a risk factor for respiratory admission among population, especially among children aged < 5 years old and the ozone effect was different between winter and summer season. Further studies on the effects of other air pollutants and their interaction on paediatric admission in Hanoi should be considered.

## Supporting information

S1 Table Associations between O_3_ and hospital admissions for respiratory diseases (Non threshold approach). RR: risk ratio, CI: confidence interval.
(DOCX)

S2 Table Associations between O_3_ and hospital admissions for wheeze-associated disorders (Non threshold approach). RR: risk ratio, CI: confidence interval.
(DOCX)

S3 Table Associations between O_3_ and hospital admissions (Threshold approach). RR: risk ratio, CI: confidence interval.
(DOCX)
