# Supplementary material for: Seasonal association between ambient ozone and hospital admission for respiratory diseases in Hanoi, Vietnam
Source: PLoS One. 2018 Sep 24;13(9):e0203751. doi: 10.1371/journal.pone.0203751 (PMC6152873; doi:10.1371/journal.pone.0203751)
Supplement: S1 Table — RR: risk ratio, CI: confidence interval. (DOCX) [file pone.0203751.s001.docx]

**Seasonal association between ambient ozone and hospital admission for respiratory diseases in Hanoi, Vietnam**

Ly M. T. Luong^1,2,3^, Dung Phung^4*^_,_ Tran Ngoc Dang^5,7^, Peter D. Sly^1,2^, Lidia Morawska^6^, Phong K. Thai^6*^

^1^Faculty of Medicine, The University of Queensland, Brisbane, Australia

^2^Children's Health and Environment Program, The University of Queensland, Brisbane, Australia

^3^Faculty of Environmental Sciences, VNU University of Science, Hanoi, Vietnam

^4^Centre for Environment and Population Health, Griffith University, Brisbane, Australia

^5^The Institute of Research and Development, Duy Tan University, Da Nang, Vietnam

^6^International Laboratory for Air Quality & Health, Queensland University of Technology, Brisbane, Australia

^7^Department of Environmental Health, University of Medicine and Pharmacy at Ho Chi Minh City, Vietnam

*Corresponding authors:

Phong K. Thai, Email address: [phong.thai@qut.edu.au](mailto:phong.thai@qut.edu.au)

Dung Phung, Email address: [d.phung@griffith.edu.au](mailto:d.phung@griffith.edu.au)

**SUPPORTING INFORMATION**

**S1 Table.** **Associations between O_3_ and hospital admissions for respiratory diseases (Non threshold approach)**

| Age | Lag | Association with a 10 µg/m^3^ increase in O_3_ | | | | | | | | | | |  |
| --- | --- | --- | --- | --- | --- | --- | --- | --- | --- | --- | --- | --- | --- |
|  |  | Full year | | |  | Winter | | |  | Summer | | | |
|  |  | RR | 95% CI | |  | RR | 95% CI | |  | RR | 95% CI | |  |
|  |  |  | Lower | Upper |  |  | Lower | Upper |  |  | Lower | Upper | |
| All ages | Overall | 1.007 | 1.001 | 1.013 |  | 1.056 | 1.034 | 1.079 |  | 1.012 | 1.004 | 1.02 | |
|  | Lag 0 | 0.999 | 0.994 | 1.003 |  | 1.000 | 0.982 | 1.018 |  | 0.999 | 0.993 | 1.006 | |
|  | Lag 1 | 1.001 | 0.996 | 1.006 |  | 1.015 | 0.997 | 1.033 |  | 1.002 | 0.995 | 1.009 | |
|  | Lag 2 | 1.003 | 0.998 | 1.008 |  | 1.005 | 0.987 | 1.023 |  | 1.004 | 0.997 | 1.011 | |
|  | Lag 3 | 1.006 | 1.001 | 1.011 |  | 1.011 | 0.994 | 1.029 |  | 1.007 | 1.000 | 1.014 | |
|  | Lag 4 | 1.000 | 0.995 | 1.005 |  | 1.005 | 0.988 | 1.023 |  | 0.999 | 0.992 | 1.006 | |
|  | Lag 5 | 0.998 | 0.994 | 1.003 |  | 1.019 | 1.003 | 1.036 |  | 1.001 | 0.995 | 1.008 | |
| <5 years old | Overall | 1.007 | 1.000 | 1.013 |  | 1.062 | 1.037 | 1.088 |  | 1.012 | 1.003 | 1.021 | |
|  | Lag 0 | 1.000 | 0.995 | 1.006 |  | 1.001 | 0.981 | 1.021 |  | 1.000 | 0.993 | 1.008 | |
|  | Lag 1 | 1.001 | 0.995 | 1.007 |  | 1.016 | 0.996 | 1.037 |  | 1.002 | 0.994 | 1.010 | |
|  | Lag 2 | 1.001 | 0.995 | 1.006 |  | 1.008 | 0.988 | 1.028 |  | 1.003 | 0.995 | 1.011 | |
|  | Lag 3 | 1.006 | 1.001 | 1.012 |  | 1.008 | 0.988 | 1.028 |  | 1.005 | 0.997 | 1.013 | |
|  | Lag 4 | 0.998 | 0.992 | 1.004 |  | 1.004 | 0.984 | 1.024 |  | 0.999 | 0.991 | 1.007 | |
|  | Lag 5 | 1.000 | 0.995 | 1.005 |  | 1.025 | 1.007 | 1.044 |  | 1.003 | 0.995 | 1.010 | |
| > 65 years old | Overall | 1.021 | 1.005 | 1.037 |  | 1.024 | 0.977 | 1.073 |  | 0.993 | 0.969 | 1.019 | |
|  | Lag 0 | 0.996 | 0.983 | 1.008 |  | 0.971 | 0.932 | 1.011 |  | 0.981 | 0.962 | 1.001 | |
|  | Lag 1 | 1.005 | 0.991 | 1.018 |  | 1.030 | 0.991 | 1.072 |  | 1.002 | 0.981 | 1.024 | |
|  | Lag 2 | 1.006 | 0.992 | 1.019 |  | 0.988 | 0.950 | 1.028 |  | 1.008 | 0.986 | 1.031 | |
|  | Lag 3 | 1.011 | 0.998 | 1.024 |  | 1.022 | 0.984 | 1.062 |  | 1.014 | 0.992 | 1.036 | |
|  | Lag 4 | 1.005 | 0.992 | 1.019 |  | 1.028 | 0.988 | 1.07 |  | 0.995 | 0.973 | 1.018 | |
|  | Lag 5 | 0.999 | 0.987 | 1.011 |  | 0.986 | 0.951 | 1.022 |  | 0.993 | 0.972 | 1.014 | |

_RR: risk ratio, CI: confidence interval_
