# Supplementary material for: Seasonal association between ambient ozone and hospital admission for respiratory diseases in Hanoi, Vietnam
Source: PLoS One. 2018 Sep 24;13(9):e0203751. doi: 10.1371/journal.pone.0203751 (PMC6152873; doi:10.1371/journal.pone.0203751)
Supplement: S3 Table — RR: risk ratio, CI: confidence interval. (DOCX) [file pone.0203751.s003.docx]

**Seasonal association between ambient ozone and hospital admission for respiratory diseases in Hanoi, Vietnam**

Ly M. T. Luong^1,2,3^, Dung Phung^4*^_,_ Tran Ngoc Dang^5,7^, Peter D. Sly^1,2^, Lidia Morawska^6^, Phong K. Thai^6*^

^1^Faculty of Medicine, The University of Queensland, Brisbane, Australia

^2^Children's Health and Environment Program, The University of Queensland, Brisbane, Australia

^3^Faculty of Environmental Sciences, VNU University of Science, Hanoi, Vietnam

^4^Centre for Environment and Population Health, Griffith University, Brisbane, Australia

^5^The Institute of Research and Development, Duy Tan University, Da Nang, Vietnam

^6^International Laboratory for Air Quality & Health, Queensland University of Technology, Brisbane, Australia

^7^Department of Environmental Health, University of Medicine and Pharmacy at Ho Chi Minh City, Vietnam

*Corresponding authors:

Phong K. Thai, Email address: [phong.thai@qut.edu.au](mailto:phong.thai@qut.edu.au)

Dung Phung, Email address: [d.phung@griffith.edu.au](mailto:d.phung@griffith.edu.au)

**SUPPORTING INFORMATION**

**S3 Table. Associations between O_3_ and hospital admissions** (Threshold approach)

| Diseases | Age | Lag | Association with a 10 µg/m^3^ increase in O_3_ above threshold | | | | | | |
| --- | --- | --- | --- | --- | --- | --- | --- | --- | --- |
|  |  |  | Winter (threshold =42.4 µg/m^3^ ) | | |  | Summer (threshold =52 µg/m^3^) | | |
|  |  |  | RR | 95% CI | |  | RR | 95% CI | |
|  |  |  |  | Lower | Upper |  |  | Lower | Upper |
| All causes of respiratory diseases | All ages | Overall | 1.062 | 1.031 | 1.093 |  | 1.015 | 1.005 | 1.026 |
|  |  | Lag 0 | 0.994 | 0.970 | 1.018 |  | 0.998 | 0.991 | 1.006 |
|  |  | Lag 1 | 1.010 | 0.984 | 1.036 |  | 1.002 | 0.994 | 1.010 |
|  |  | Lag 2 | 1.017 | 0.991 | 1.043 |  | 1.004 | 0.997 | 1.012 |
|  |  | Lag 3 | 1.014 | 0.989 | 1.039 |  | 1.010 | 1.002 | 1.018 |
|  |  | Lag 4 | 0.990 | 0.966 | 1.015 |  | 0.999 | 0.991 | 1.007 |
|  |  | Lag 5 | 1.036 | 1.013 | 1.060 |  | 1.001 | 0.994 | 1.009 |
|  |  |  |  |  |  |  |  |  |  |
|  | <5 years old | Overall | 1.073 | 1.037 | 1.109 |  | 1.017 | 1.006 | 1.028 |
|  |  | Lag 0 | 0.995 | 0.966 | 1.024 |  | 1.000 | 0.992 | 1.009 |
|  |  | Lag 1 | 1.020 | 0.990 | 1.051 |  | 1.002 | 0.993 | 1.012 |
|  |  | Lag 2 | 1.015 | 0.986 | 1.045 |  | 1.004 | 0.995 | 1.014 |
|  |  | Lag 3 | 1.011 | 0.983 | 1.040 |  | 1.007 | 0.998 | 1.016 |
|  |  | Lag 4 | 0.995 | 0.967 | 1.023 |  | 0.998 | 0.989 | 1.007 |
|  |  | Lag 5 | 1.036 | 1.010 | 1.063 |  | 1.005 | 0.996 | 1.014 |
|  |  |  |  |  |  |  |  |  |  |
|  | > 65 years old | Overall | 1.025 | 0.959 | 1.095 |  | 1.014 | 0.984 | 1.046 |
|  |  | Lag 0 | 0.976 | 0.920 | 1.035 |  | 0.987 | 0.964 | 1.010 |
|  |  | Lag 1 | 1.040 | 0.981 | 1.103 |  | 1.001 | 0.977 | 1.026 |
|  |  | Lag 2 | 0.984 | 0.927 | 1.044 |  | 1.010 | 0.986 | 1.036 |
|  |  | Lag 3 | 1.023 | 0.968 | 1.081 |  | 1.022 | 0.998 | 1.047 |
|  |  | Lag 4 | 1.016 | 0.959 | 1.076 |  | 0.996 | 0.971 | 1.022 |
|  |  | Lag 5 | 0.988 | 0.936 | 1.042 |  | 0.998 | 0.974 | 1.022 |
|  |  |  |  |  |  |  |  |  |  |
| Wheeze-associated disorders | All ages | Overall | 1.038 | 0.970 | 1.110 |  | 0.967 | 0.935 | 1.000 |
|  |  | Lag 0 | 0.962 | 0.910 | 1.017 |  | 1.002 | 0.975 | 1.029 |
|  |  | Lag 1 | 1.037 | 0.979 | 1.098 |  | 0.996 | 0.966 | 1.027 |
|  |  | Lag 2 | 1.009 | 0.953 | 1.069 |  | 0.986 | 0.956 | 1.017 |
|  |  | Lag 3 | 1.011 | 0.957 | 1.069 |  | 1.003 | 0.973 | 1.034 |
|  |  | Lag 4 | 0.997 | 0.942 | 1.055 |  | 0.991 | 0.961 | 1.023 |
|  |  | Lag 5 | 1.023 | 0.971 | 1.077 |  | 0.988 | 0.960 | 1.017 |
|  |  |  |  |  |  |  |  |  |  |
|  | < 5 years old | Overall | 1.032 | 0.943 | 1.129 |  | 0.957 | 0.917 | 0.998 |
|  |  | Lag 0 | 0.962 | 0.888 | 1.042 |  | 1.014 | 0.981 | 1.047 |
|  |  | Lag 1 | 1.046 | 0.968 | 1.129 |  | 0.983 | 0.946 | 1.022 |
|  |  | Lag 2 | 0.994 | 0.922 | 1.071 |  | 0.996 | 0.959 | 1.034 |
|  |  | Lag 3 | 1.035 | 0.964 | 1.113 |  | 0.996 | 0.959 | 1.035 |
|  |  | Lag 4 | 0.956 | 0.890 | 1.027 |  | 0.978 | 0.940 | 1.016 |
|  |  | Lag 5 | 1.043 | 0.977 | 1.113 |  | 0.990 | 0.956 | 1.025 |
|  |  |  |  |  |  |  |  |  |  |
|  | > 65 years old | Overall | 1.080 | 0.866 | 1.347 |  | 0.982 | 0.876 | 1.101 |
|  |  | Lag 0 | 1.033 | 0.854 | 1.249 |  | 0.918 | 0.828 | 1.018 |
|  |  | Lag 1 | 0.981 | 0.806 | 1.193 |  | 1.066 | 0.975 | 1.165 |
|  |  | Lag 2 | 1.002 | 0.832 | 1.206 |  | 0.956 | 0.864 | 1.059 |
|  |  | Lag 3 | 1.059 | 0.885 | 1.268 |  | 0.976 | 0.882 | 1.080 |
|  |  | Lag 4 | 0.936 | 0.775 | 1.130 |  | 1.037 | 0.943 | 1.141 |
|  |  | Lag 5 | 1.074 | 0.908 | 1.270 |  | 1.037 | 0.950 | 1.132 |
|  |  |  |  |  |  |  |  |  |  |

_RR: risk ratio, CI: confidence interval_
